# Capsaicin-sensitive primary sensory neurons in the mouse express *N*-Acyl phosphatidylethanolamine phospholipase D

**DOI:** 10.1016/j.neuroscience.2009.03.047

**Published:** 2009-06-30

**Authors:** B. Nagy, C. Fedonidis, A. Photiou, J. Wahba, C.C. Paule, D. Ma, L. Buluwela, I. Nagy

**Affiliations:** a Department of Anaesthetics, Pain Medicine and Intensive Care, Imperial College London, Faculty of Medicine, Chelsea and Westminster Hospital, 369 Fulham Road, London SW10 9NH, UK; b Department of Oncology, Imperial College London, Hammersmith Hospital, Du Cane Road, London W12 0NN, UK

**Keywords:** anandamide, dorsal root ganglion, transient receptor vanilloid type 1, TRPV1, nociceptive, pain, anandamide, N-arachidonoyl ethanolamine, CB1, cannabinoid 1, CB2, cannabinoid 2, DRG, dorsal root ganglia, FAAH, fatty acid amide hydrolase, GAPDH, glyceraldehyde-3-phosphate dehydrogenase, NAPE-PLD, *N*-acyl phosphotidylethanolamine phospholipase D, PCR, polymerase chain reaction, RT, reverse transcriptase, TRPV1, transient receptor potential vanilloid type 1

## Abstract

Our previous finding, that the capsaicin- and KCl-induced Ca^2+^-dependent production of the intra- and intercellular signaling molecule *N*-arachidonoyl ethanolamine (anandamide) in cultured primary sensory neurons could be abolished and reduced by ∼2/3 by capsaicin-induced degeneration of capsaicin-sensitive neurons, respectively suggests that a major sub-population of capsaicin-sensitive cells together with a group of non-capsaicin-sensitive cells should express enzymes involved in Ca^2+^-dependent anandamide synthesis. *N*-acyl phosphotidylethanolamine phospholipase D (NAPE-PLD) is known to be involved in Ca^2+^-dependent anandamide production. Hence, here, we used reverse transcriptase and quantitative real time polymerase chain reaction to study NAPE-PLD expression in dorsal root ganglia and to clarify the sub-population of cells expressing this enzyme. Cultures prepared from mouse dorsal root ganglia were grown either in the absence or presence of the neurotoxin, capsaicin (10 μM) overnight. We report, that NAPE-PLD is expressed both in dorsal root ganglia and cultures prepared from dorsal root ganglia and grown in the absence of capsaicin. Furthermore, we also report that capsaicin application downregulates the expression of NAPE-PLD as well as the capsaicin receptor, transient receptor potential vanilloid type 1 ion channel, by about 70% in the cultures prepared from dorsal root ganglia. These findings indicate that a major sub-population of capsaicin-sensitive primary sensory neurons expresses NAPE-PLD, and suggest that NAPE-PLD is expressed predominantly by capsaicin-sensitive neurons in dorsal root ganglia. These data also suggest that NAPE-PLD might be a target to control the activity and excitability of a major sub-population of nociceptive primary sensory neurons.

The great majority of nociceptive primary sensory neurons are sensitive to capsaicin, the agent that is responsible for the hotness of the chili pepper (Porszasz and Jancso, 1959; Jancso et al., 1977; Lynn and Carpenter, 1982; Heyman and Rang, 1985; Wood et al., 1988). Capsaicin-sensitivity of cells is underlain by the expression of the capsaicin receptor, transient receptor potential vanilloid type 1 ion channel (TRPV1; Caterina et al., 1997, 2000; Davis et al., 2000). In addition to capsaicin, TRPV1 is also responsive to a series of other activators such as moderate heat, protons, post-translational modifications, depolarization and other exogenous and endogenous agents, including *N*-arachidonoyl ethanolamine (anandamide; Caterina et al., 1997; Zygmunt et al., 1999; Huang et al., 2002; Shin et al., 2002; Chu et al., 2003; Voets et al., 2004; Movahed et al., 2005). Anandamide, in addition to TRPV1, is also an activator of other receptors, including the cannabinoid 1 (CB1) and cannabinoid 2 (CB2) receptors, and the orphan G protein-coupled receptor 55 (GPR 55) (Matsuda et al., 1990; Devane et al., 1992; Munro et al., 1993; Zygmunt et al., 1999; Ryberg et al., 2007). Of these, the CB1 and CB2 receptors are co-expressed with TRPV1 in a major sub-population of primary sensory neurons (Ahluwalia et al., 2000; Ross et al., 2001; Agarwal et al., 2007; Anand et al., 2008). Previous findings suggest that anandamide, by activating TRPV1 and the cannabinoid receptors, may be involved in the regulation of the activity and excitability of the TRPV1/CB1 receptor-expressing cells (Ellington et al., 2002; Ahluwalia et al., 2003a; Németh et al., 2003; Anand et al., 2008).

Interestingly, primary sensory neurons, including the capsaicin-sensitive cells are capable of producing anandamide (Ahluwalia et al., 2003b; van der Stelt et al., 2005; Vellani et al., 2008). Anandamide-production in primary sensory neurons could depend on, or could be independent of, Ca^2+^ (Ahluwalia et al., 2003b; van der Stelt et al., 2005; Vellani et al., 2008). The Ca^2+^-dependent anandamide production could be triggered by capsaicin, KCl-induced depolarization or by Ca^2+^ release from the intracellular stores (Ahluwalia et al., 2003b; van der Stelt et al., 2005). While the capsaicin-induced anandamide production is completely abolished, the KCl-induced anandamide synthesis is reduced to about one third of the control value, by overnight capsaicin treatment, which induces Ca^2+^-dependent cytotoxicity and cellular death (Jancso et al., 1977, 1995; Gamse et al., 1982; Chard et al., 1995; Wood et al., 1988; Olah et al., 2001; Ahluwalia et al., 2003b).

The enzyme, *N*-acyl phosphotidylethanolamine phospholipase D (NAPE-PLD), belongs to the zinc metallohydrolase family of the β-lactamase fold (Okamoto et al., 2004) and is known to produce various long and medium chain bioactive *N*-acylethanolamines, including anandamide in a Ca^2+^-dependent fashion (Okamoto et al., 2004; Sun et al., 2004; Leung et al., 2006; Liu et al., 2006; Simon and Cravatt, 2006, 2008). Therefore, we hypothesized that NAPE-PLD could be expressed by a major sub-population of capsaicin-sensitive cells in addition to a group of non-capsaicin-sensitive cells, which could belong to the non-nociceptive sub-population of primary sensory neurons. Accordingly, here, we studied the expression of NAPE-PLD in dorsal root ganglia (DRG) and cultures prepared from DRG. In order to find out whether NAPE-PLD is expressed by the capsaicin-sensitive cells, half of the cultures were grown in the presence of capsaicin.

## Experimental procedures

### Animals and preparation of primary sensory neuronal cultures

All procedures in this work were performed in accordance with the UK Animals (Scientific Procedures) Act 1986, and its ethical guidelines and every effort was taken to minimize the number and suffering of animals used. Measurements were performed on tissue homogenates of DRG, heart and total brain, collected from 10- to 12-week-old male C57BL/6 mice and on cultures, which were prepared from DRG collected also from 10- to 12-week-old male C57BL/6 mice.

Tissues were quickly dissected out from terminally anesthetized (Enflurane; Abbott Laboratories, Kent, UK) animals and chopped into small pieces in RNAlater (QIAGEN, Crawley, UK). Tissue samples were stored in RNAlater at 4 °C until they were used for RNA isolation.

Cultures were prepared following terminal anesthesia (Enflurane, Abbott Laboratories) as described previously (Nagy and Rang, 1999). Briefly, ganglia were removed from the first cervical to the first sacral segment from both sides and placed into Dulbecco's Modified Eagle's Medium F12 (Sigma, Gillingham, UK) supplemented with 2 mM *l-*glutamine (Invitrogen, Paisley, UK), 5000 IU/ml penicillin (Invitrogen, Paisley, UK), 5000 μg/ml streptomycin (Invitrogen, Paisley, UK) and 2% Ultroser G (BioSepra SA, Cergy-Pontoise, France). Connective tissues in the DRG were digested by 0.125% type IV collagenase (Lorne Diagnostics, Bury St. Edmunds, UK) for 3 h at 37 °C in 5% CO_2_. Ganglia were triturated with a fire-polished Pasteur pipette, and cells were plated on poly dl-ornithine-coated glass coverslips (Sigma). Cells were cultured at 37 °C in a humidified atmosphere gassed with 5% CO_2_ for a day in the supplemented F12 medium to which 50 ng/ml nerve growth factor was added (Promega, Southampton, UK). Half of the cultures prepared from three mice were grown in the presence of 10 μM capsaicin, which was dissolved in dimethyl sulfoxide (final concentration 3 mM).

### Isolation of mRNA and reverse transcriptase (RT) reaction

Tissue samples were weighed and homogenized by a tissue homogenizer. Cell lysates were further homogenized using QIAshredder columns (QIAGEN, Crawley, UK). Cultured cells were scraped from the coverslips and homogenized using QIAshredder columns. RNA from the homogenates was extracted using the RNeasy Mini Kit (QIAGEN) according to the manufacturer's instructions. Following elution the RNA was quantified and stored at −80 °C until further use.

Total RNA (600 ng) was reverse transcribed with SuperScript II (Invitrogen), using oligo (dT)_15_ primer (Promega), dNTP (Promega), SUPERasin (Ambion, Huntington, UK), first-strand buffer (Invitrogen) and DTT (Invitrogen).

### Polymerase chain reaction (PCR)

Primers were designed to amplify mouse NAPE-PLD and the housekeeping gene, glyceraldehyde-3-phosphate dehydrogenase (GAPDH). The sequences of the primers (MWG Biotech, Ebersberg, Germany) were as follows: NAPE-PLD: forward 5′-GGC CAA CAT GGA AAA ACA TC-3′; reverse: 5′-ATG AGC TCG TCC ATT TCC AC-3′; GAPDH: forward: 5′-GGT GAA GGT CGG AGT CAA CG-3′; reverse: 5′-CAA AGT TGT CAT GGA TTG ACC-3′. The predicted product sizes were 222 and 370 bp for NAPE-PLD and GAPDH, respectively. The PCR reaction mixture contained 3 mM MgCl_2_, 1× reaction buffer (5 mM Tris–HCl, 50 mM KCl, 1.5 mM MgCl_2_, pH 8.3), 0.2 mM deoxynucleotide mix and 1.25 U of Go-Taq Flexi DNA polymerase (Promega). The amplification reaction consisted of 30 cycles with 30 s of denaturation at 96 °C, 1 min annealing, and 3 min extension at 72 °C in a thermal cycler (Eppendorf-Mastercycler Personal, UK). The annealing temperatures for both NAPE-PLD and GAPDH were 55 °C. PCR products were separated on agarose gels and visualized with ethidium bromide.

### Quantitative real time PCR

For quantitative real-time PCR, specific assays were obtained (PrimerDesign, Ltd., UK). The primers were designed and validated by the manufacturer. In these experiments we assessed the effect of capsaicin treatment on the expression of NAPE-PLD and TRPV1 relative to the expression of GAPDH. The primers for TRPV1 were designed against the GenBank sequence NM_001001445 and were as follows; forward: 5′-CCT GCA TTG ACA CCT GTG AA-3′; reverse: 5′-AGT CGG TTC AAG GGT TCC A-3′. Primers for NAPE-PLD were designed using GenBank sequence NM_178728: forward: 5′-GGG CGG CTC TCA CTT TCT A-3; reverse: 5′-ACA CTT GTG CTT ATA GGT CAT TTA AT-3′. For GAPDH a pre-designed primer set was provided by the manufacturer. The reaction was performed in triplicate using Precision qPCR master mix with SYBR green and ROX (PrimerDesign, Ltd.) on an ABI 7900HT real-time PCR machine. These reactions were enzyme-activated by heating at 95 °C for 10 min (hot start), then denaturing at 95 °C for 15 s followed by cooling to 60 °C for data collection (50 cycles). The “crossover” threshold (ct) was determined in each sample for each DNA. The average GAPDH measurement in each sample was used to establish the relative expression of NAPE-PLD and TRPV1 in the respective sample.

## Results

First, we aimed to establish whether NAPE-PLD is expressed in DRG and in cultures prepared from DRG. In addition to cDNA from DRG and cultures, cDNA from the heart, where NAPE-PLD has been cloned from (Okamoto et al., 2004), and the brain, where NAPE-PLD expression has been reported recently (Morishita et al., 2005; Leung et al., 2006), was also included in the reaction, for control.

**Fig. 1:**
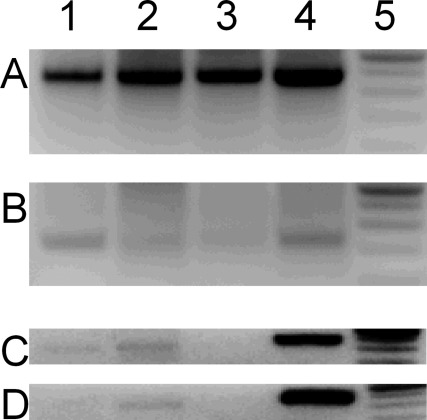
RT-PCR analysis of NAPE-PLD gene expression. (A) Agarose gel electrophoresis of RT-PCR products for GAPDH (370 bp) from cDNA made to RNA from brain, heart, DRG and cultures prepared from DRG (lanes 1, 2, 3, and 4, respectively). (B) Agarose gel electrophoresis of RT-PCR products for NAPE-PLD (222 bp) from cDNA made to RNA from brain, heart, DRG and cultures prepared from DRG (lanes 1, 2, 3, and 4, respectively). Note that NAPE-PLD is expressed in all the tissues we examined. (C) NAPE-PLD (lane 1) and GAPDH (lane 4) gene expression in mouse dorsal root ganglion cultures grown under control conditions (without capsaicin). Lane 2 shows NAPE-PLD expression in a brain sample collected from the same animal used to derive the cultures analyzed in lanes 1 and 4. Lane 3 is a control PCR reaction, where RNA equivalent to the cDNA used in lane 1 has been used as template. (D) NAPE-PLD (lane 1) and GAPDH (lane 4) gene expression in mouse dorsal root ganglion cultures grown in the presence of 10 μM capsaicin overnight. Lane 2 shows NAPE-PLD expression in the brain sample collected from the same animal. Lane 3 is a control PCR reaction, where RNA equivalent to the cDNA used in lane 1 has been used as template. Note that the treatment of cultures with capsaicin significantly downregulated NAPE-PLD expression (lane 1 in C and D.)

RT-PCR produced distinct products with sizes between 350 and 400 bp, and 200 and 250 bp in all samples (Fig. 1A, B). While the larger product (Fig. 1A) corresponded with the predicted size of the GAPDH, the smaller product (Fig. 1B) corresponded with that expected for the NAPE-PLD RT-PCR product. These findings indicated that NAPE-PLD is expressed both in DRG and cultures prepared from DRG.

In addition to primary sensory neurons, both DRG and cultures prepared from DRG contain cells other then primary sensory neurons. However, only a sub-population of primary sensory neurons is susceptible to degeneration by the neurotoxin, capsaicin in both intact DRG and cultures prepared from DRG (Porszasz and Jancso, 1959; Jancso et al., 1977; Lynn and Carpenter, 1982; Heyman and Rang, 1985; Wood et al., 1988). Thus, in order to find out the cell type expressing NAPE-PLD, we induced capsaicin-evoked degeneration of capsaicin-sensitive cells by growing the cultures in 10 μM of this neurotoxin overnight (Gamse et al., 1982; Chard et al., 1995; Jancso et al., 1995). In agreement with results of the previous experiment, RT-PCR produced NAPE-PLD amplicon when cDNA of control cultures served as a template (Fig. 1C, lane 1). However, very little PCR product was detected when cDNA from the capsaicin-treated cultures was used as a template for the reaction (Fig. 1D, lane 1).

**Fig. 2:**
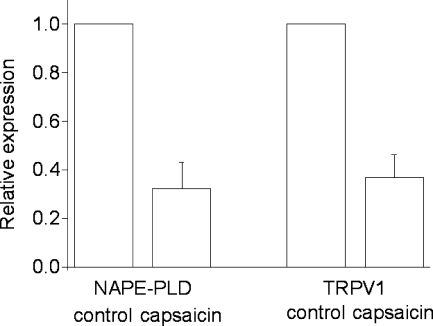
Relative gene expression of NAPE-PLD and TRPV1 in cultures prepared from DRG and grown either in the absence (control) or presence of 10 μM capsaicin overnight. The expression of the NAPE-PLD and TRPV1 mRNA was normalized to that of GAPDH mRNA. Each set shows an average from three cultures, each prepared from different animals, with standard errors of the mean shown. Note that the treatment of cultures with capsaicin downregulated both TRPV1 and NAPE-PLD-expression relative to that seen in untreated cultures.

In order to ascertain that capsaicin treatment indeed downregulated NAPE-PLD expression, we next measured the relative expression of NAPE-PLD, and TRPV1, which mediates the capsaicin-induced neurotoxic Ca^2+^ influx (Caterina et al., 1997), in control and capsaicin-treated cultures with real time quantitative PCR. We found that capsaicin treatment reduced NAPE-PLD mRNA expression by about 70% (Fig. 2). The capsaicin treatment downregulated TRPV1 mRNA expression by the same extent.

## Discussion

At present, TRPV1 is the only molecule, which is known to selectively and specifically respond to capsaicin (Caterina et al., 1997, 2000; Davis et al., 2000). Immunohistochemical and functional data show that only a sub-population of neurons expresses TRPV1 in DRG and cultures prepared from DRG (Nagy et al., 1993; Caterina et al., 1997, 2000; Guo et al., 1999; Michael and Priestley, 1999; Ahluwalia et al., 2000; Singh Tahim et al., 2005). Thus, capsaicin treatment can induce degeneration only in the TRPV1-expressing sub-population of neurons in cultures prepared from DRG.

We found in the present study that overnight exposure of cultured primary sensory neurons to capsaicin reduced TRPV1 mRNA expression by ∼70%. This degree of downregulation in TRPV1 mRNA expression is comparable with the ∼70%–80% reduction in TRPV1 protein expression and in the number of TRPV1-expressing neurons produced by prolonged (≥48 h) application of the ultrapotent TRPV1 activator, resiniferatoxin to primary sensory neurons (Olah et al., 2001; Tender et al., 2005). In parallel with the reduction in TRPV1 mRNA expression, NAPE-PLD mRNA expression was also reduced by ∼70%. These findings indicate that a major proportion of capsaicin-sensitive primary sensory neurons expresses NAPE-PLD, and suggest that the majority of the NAPE-PLD-expressing primary sensory neurons are capsaicin sensitive. However, assuming that the TRPV1 mRNA we detected in our cultures following the capsaicin treatment, was expressed in neurons, which, in spite of their TRPV1 expression, were not responsive to capsaicin, or alternatively, they were responsive to capsaicin but resistant to the subsequent excitotoxicity, it is tempting to propose that TRPV1 and NAPE-PLD could be co-expressed to a high degree, in primary sensory neurons.

Based on the similarity between the overnight 10 μM capsaicin exposure-evoked downregulation of NAPE-PLD mRNA expression, and the previously demonstrated downregulation in KCl-evoked anandamide release (∼60%–70%; Ahluwalia et al., 2003b), it is also tempting to propose that only NAPE-PLD could be responsible for Ca^2+^-dependent anandamide production in primary sensory neurons. However, in addition to the Ca^2+^-evoked anandamide synthesis (Ahluwalia et al., 2003b; van der Stelt et al., 2005), primary sensory neurons, also produce anandamide in a Ca^2+^-independent manner (Vellani et al., 2008). Therefore, NAPE-PLD must represent only one of the anandamide-synthesizing enzymatic pathways present in these cells. To the best of our knowledge, this is the first report demonstrating the expression of an enzyme that is involved in anandamide synthesis in primary sensory neurons, and it is not known which other anandamide-producing enzymatic pathways are expressed, and whether any of the other pathways are involved in anandamide synthesis in a Ca^2+^-dependent fashion, in these neurons (Di Marzo and Petrosino, 2007). Therefore, further studies are needed to elucidate these issues.

Nevertheless, the presence of NAPE-PLD in capsaicin-sensitive primary sensory neurons raises the question, what role does this enzyme play in these neurons. Several of the NAPE-PLD products, including oleoylethanolamide, linoleoylethanolamide and anandamide, activate TRPV1 (Zygmunt et al., 1999; Okamoto et al., 2004; Movahed et al., 2005). However, the finding that among these products, anandamide also activates a series of inhibitory receptors, which are co-expressed with TRPV1 in primary sensory neurons (Matsuda et al., 1990; Devane et al., 1992; Munro et al., 1993; Zygmunt et al., 1999; Ahluwalia et al., 2000; Ross et al., 2001; Agarwal et al., 2007; Anand et al., 2008) suggests that anandamide production by NAPE-PLD could be the most important in relation to regulating the activity and excitability of a major sub-population of capsaicin-sensitive, thus, nociceptive cells.

The expression pattern of the anandamide-responding receptors in nociceptive primary sensory neurons (Ahluwalia et al., 2000; Ross et al., 2001; Agarwal et al., 2007; Anand et al., 2008) together with previous functional data (Ellington et al., 2002; Ahluwalia et al., 2003b; Németh et al., 2003; Nagy et al., 2006; Anand et al., 2008) suggests that NAPE-PLD activity may provide a CB1- and/or CB2-mediated brake on the responsiveness and activity of, the cells, and TRPV1. Alternatively, NAPE-PLD might be part of a signal amplification pathway in TRPV1-expressing cells, which has been suggested recently by van der Stelt and Di Marzo (2005). The presence of such an amplification system in capsaicin-sensitive primary sensory neurons is supported by recent findings. First, van der Stelt and co-workers (2005) have reported that the Ca^2+^-dependent anandamide production results in TRPV1 activity in cultured primary sensory neurons. Second, we found that inhibition of the anandamide-hydrolysing enzyme, fatty acid amide hydrolase (FAAH), which is also expressed by a major sub-population of TRPV1-expressing primary sensory neurons results in TRPV1 activity (Lever et al., 2009). Third, repeated application of anandamide to TRPV1 sensitizes the responses of this ion channel (Premkumar and Ahern, 2000).

In primary sensory neurons, TRPV1 seems to have a pivotal role in signaling peripheral inflammatory events to the CNS through getting activated directly or indirectly by inflammatory mediators, which are produced and released from the inflamed tissues (Caterina et al., 2000; Davis et al., 2000; Ji et al., 2004; Ma and Quirion, 2007; Nagy et al., 2008, 2009). A series of inflammatory mediators induces Ca^2+^ influx into primary sensory neurons, including into the capsaicin-sensitive cells (Thayer et al., 1998; Cesare et al., 1999; Smith et al., 2000; Moriyama et al., 2005). Comparison of the increase in the intracellular Ca^2+^ concentration produced by some of the inflammatory mediators, including bradykinin and prostaglandin E_2_, to those which can evoke anandamide production (van der Stelt et al., 2005) suggests that inflammatory mediators should be capable of inducing anandamide production. In addition to inducing Ca^2+^ influx, inflammatory mediators also induce post-translational changes in TRPV1 (for references see Nagy et al., 2008, 2009). These changes together with the sensitizing effect of anandamide on TRPV1 (Premkumar and Ahern, 2000) result in a significant increase in the otherwise modest efficacy and potency of anandamide on TRPV1 (Zygmunt et al., 1999; Ahluwalia et al., 2003b; Singh Tahim et al., 2005). Thus, anandamide produced even in small concentrations inside the TRPV1-expressing cells can induce a significant increase in TRPV1 open probability and subsequent action potential generation. In addition to anandamide, however, the production of other TRPV1-activating N-acylethanolamines by NAPE-PLD may also contribute to TRPV1 activity in inflammatory conditions (Movahed et al., 2005).

Based on the considerations above, we propose that NAPE-PLD expression and activity in capsaicin-sensitive primary sensory neurons could serve as a vital part of the anandamide-mediated signal amplification process (van der Stelt and Di Marzo, 2005). That signal amplification, hence NAPE-PLD activity, could be fundamental for the development of increased responsiveness of these cells in pathological conditions, and subsequently, for sending information about inflammatory processes at the periphery to the CNS. However, the signal amplification process by NAPE-PLD may not be unique in capsaicin-sensitive primary sensory neurons, because increasing anandamide concentration, for example by inhibiting FAAH activity, also results in TRPV1 activity in the brain (Maione et al., 2006; Morgese et al., 2007). Thus, NAPE-PLD may also play a pivotal role in signal amplification in neurons expressing both NAPE-PLD and TRPV1 (Cristino et al., 2006, 2008), in various areas of the CNS. Nevertheless, if anandamide produced by NAPE-PLD indeed has a fundamental amplification role, which contributes to the development of TRPV1 activation and sensitization, targeting this enzyme in primary sensory neurons may provide a novel approach to reduce the activity and excitability of capsaicin-sensitive primary sensory neurons, thus, to reduce pain, in inflammatory conditions.
